# Deterministic Tractography of the Nigrostriatal-Nigropallidal Pathway in Parkinson’s Disease

**DOI:** 10.1038/srep17283

**Published:** 2015-12-01

**Authors:** Wen-Qi Tan, Chooi-Sum Yeoh, Helmut Rumpel, Nivedita Nadkarni, Weng-Kit Lye, Eng-King Tan, Ling-Ling Chan

**Affiliations:** 1 Duke-NUS Graduate Medical School, 8 College Rd, Singapore 169857; 2 Department of Diagnostic Radiology, Singapore General Hospital, Outram Rd, Singapore 169608; 3 Department of Neurology, National Neuroscience Institute, Singapore General Hospital Campus, Outram Rd, Singapore 169608.

## Abstract

We hypothesized that deterministic tractography is practical and sensitive to changes in the complex nigrostriatal and nigropallidal pathway (NSP) in Parkinson’s disease (PD). Using diffusion tensor imaging (DTI) tractography, we investigated the NSP to evaluate differences between PD patients and controls, and examined their clinico-radiologic correlation. Structural and DTI brain scans were obtained in 40 subjects (21 PD patients and 19 healthy controls). We isolated the NSP using a user-friendly DTI toolkit based on deterministic brute-force tractography. DTI parameters of fractional anisotropy (FA), mean, axial, and radial diffusivity, and streamline count of the NSP were measured. Average FA (p < 0.01) and streamline count (p < 0.001) were significantly lower in the PD compared to control group. Mean diffusivity and radial diffusivity were significantly higher in the PD group (p < 0.05). Average streamline count correlated with the United Parkinson’s Disease Rating Scale motor score (p < 0.05). Point-to-point FA profiles of the tract demonstrated peak divergence between PD and control towards the tract midpoint rather than the distal grey matter. Our findings demonstrated a clinically and radiologically practical application of DTI tractography to the NSP in PD, without requiring complex imaging sequences for anatomical localization or segmentation software.

The hallmark of Idiopathic Parkinson’s disease (PD) is severe loss of dopaminergic projection neurons of the substantia nigra (SN)^1^. The classic motor features of PD result from pathological involvement of connections from the SN to the posterior putamen and globus pallidum (GP), collectively known as the nigrostriatal and nigropallidal pathway (NSP)^2^,^3^.

Optimizing imaging methods to facilitate PD diagnosis and tracking disease progression has been a challenge. Diffusion tensor imaging (DTI) has emerged as a promising surrogate marker for PD^4^. However, data from manual region-of-interest (ROI) sampling of the SN are sometimes inconsistent^4^, and automated voxel-based analysis susceptible to registration and smoothing errors^5^. Diffusion tractography reconstructs white matter trajectories *in vivo* using computer algorithms, improving anatomical specificity^6^. These fiber pathways resolved via tractography represent indicative, rather than actual, white matter tracts. Deterministic (versus probabilistic) tractography is frequently employed in the scientific and clinical setting due to its simplicity and computation speed^7^. The streamlines generated between seed-points yield a streamline count, besides other tract-specific average DTI parameters of fractional anisotropy (FA), mean diffusivity (MD), radial diffusivity (RD), and axial diffusivity (AD).

We hypothesize that the NSP is dysregulated in PD and examining it with tractography, to obtain more data points as opposed to sampling the SN alone, could quantify neurodegeneration in PD with greater sensitivity. The potential of deterministic tractography as an alternative to probabilistic tractography also needs to be further explored given its advantages for clinical applicability. We utilized a user-friendly DTI toolkit based on deterministic brute-force tractography to evaluate for differences in the NSP pathway between PD patients and healthy individuals, and examined their clinico-radiologic correlation.

## Results

**Figure 1:**
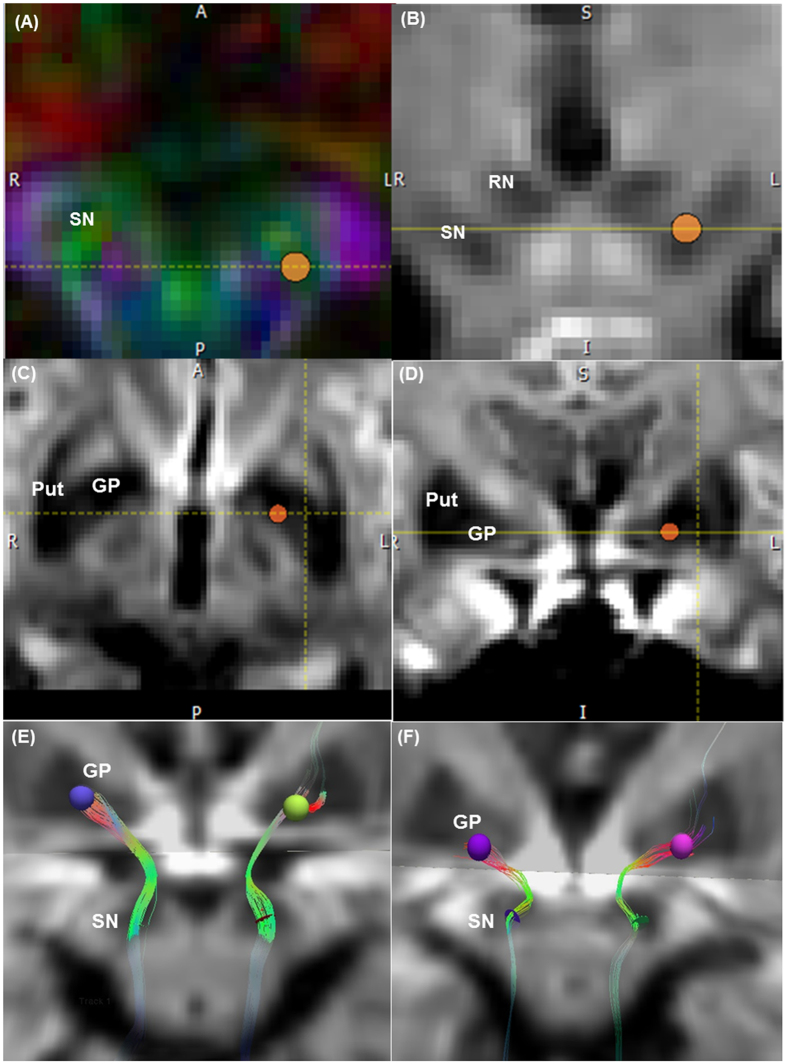
Isolating the nigrostriatal-nigropallidal tract complex on DTI. Midbrain (**A**) axial colour-FA and (**B**) coronal trace maps. First 3-mm diameter disk-shaped ROI is placed in the ventral substantia nigra (SN, green band), inferior to the level of the red nucleus (RN). Basal ganglia (**C**) axial and (**D**) coronal trace maps. Second “target” 4 mm diameter sphere ROI is placed in the ipsilateral inferomedial globus pallidus (GP). Put = putamen. Isolated nigrostriatal-nigropallidal (NSP) tract complex in a (**E**) control subject and (**F**) PD patient. The NSP tract in the PD patient was sparser than that in the control subject.

The characteristics of subjects and averaged NSP tract parameters are depicted in Table 1. Visually, the NSP tracks from PD patients were sparser than the control subjects (Fig. 1E,F) and this was reflected in the streamline count. Average FA (p = 0.002) and streamline count (p < 0.001) were lower in the PD compared to the control group. MD and RD were significantly higher in the PD group (p < 0.05). Intraclass correlation coefficient values were excellent (>0.8) for the tract measures (Table 2).

**Figure 2:**
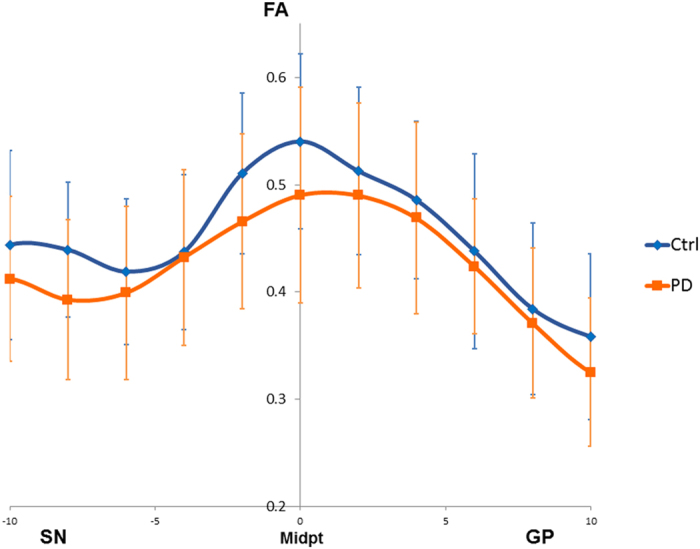
Subject-averaged FA profiles for control and Parkinson’s disease groups. The FA profiles of PD and control groups demonstrated more prominent divergence towards the middle of the tract, with the divergence peaking at the tract midpoint. SN = substantia nigra; GP = globus pallidus.

Average streamline count correlated with the UPDRS (r = −0.50, p < 0.05). Point-to-point FA profiles of the NSP tract demonstrated peak divergence between PD and control groups towards the tract midpoint rather than the distal grey matter (Fig. 2). Univariate logistic regression (table 3) showed that decreased streamline count was associated with increased odds of PD (p < 0.01), with ORs of 0.78 (95%CI 0.62 to 0.90).

## Discussion

We demonstrated a feasible application of deterministic DTI tractography to investigate the NSP pathway in PD. The tool allowed for easy localization of the SN and GP, and visualization and manipulation of the NSP tract complex. Our excellent inter-rater reliability values attest to reproducible and accurate seed-point positioning. Significant differences between PD and control in the diffusion parameters were obtained despite limited ability of deterministic tractography to resolve branching and crossing fibers. The NSP complex of PD patients had significantly lower FA than in healthy controls, and higher MD, AD and RD values, consistent with reduced fiber integrity from chronic white matter degeneration^4^,^6^. These findings, together with the reduced streamline count, an index of anatomical connectivity, point to dysregulation of the NSP pathway. Correlation of streamline count with motor disability also suggested its potential utility as a marker for clinical progression.

There was greater variation in point-to-point FA profiles towards the middle of the NSP tract compared to distal points in SN and lentiform nucleus. The findings in the lentiform nucleus are consistent with most DTI studies using manual ROI and voxel-based morphometry, which have found no difference in FA in the GP and putamen^4^,^8^. The pattern of variation along the tract lends strength to the argument that tractography may be able to identify diffusion changes in the NSP pathway in PD even when there are no detectable diffusion changes in the SN and basal ganglia.

Previous tractography studies in PD that included the NSP tracts used the FMRIB software library (FSL) for image processing, segmentation of subcortical structures, and probabilistic tractography^9^,^10^. However, decreased anatomic connectivity in the nigrostriatal and nigropallidal tracts of PD patients was detected only with additional complex imaging and software for volume segmentation of the SN^9^. The complexity of the software and protocols used pose a significant hurdle for integration in the clinical setting, and encouraging their use amongst clinicians. Our simple approach used deterministic tractography in a user friendly toolkit with readily visualized radiological landmarks to investigate the NSP pathway. The non-invasiveness of DTI is another significant advantage over positron emission tomography and other functional neuroimaging techniques.

Our results are strongly consistent with the observations by Zhang *et al.*^11^ even though the current study has used a cohort of advanced PD patients compared to early stage PD. This suggests that the impairment of DTI integrities remains significant in advanced stage PD, and DTI of the NSP could be a useful marker for PD diagnosis. Other studies suggest that duration and stage of disease may decrease the FA difference between PD and control^12^,^13^, possibly due to secondary changes in the SN such as iron deposition.

Limitations of our study include a small sample size that was insufficient to run multiple regression models accounting for age and gender, although our PD and control groups are age- and gender-matched. Additionally, diffusion tractography cannot distinguish the functional directionality of fibre tracts, and the tracts isolated may include both anterograde nigrostriatal-nigropallidal pathways and retrograde pallidonigral/thalamonigral pathways. The degree to which the latter is affected in PD is unclear, as its effect on the DTI results.

Subjects were scanned under dopaminergic medication. While this reduced movement artefact, which causes severe and significant inaccuracies to DTI measurements, the influence of dopaminergic medications on DTI results is poorly understood and requires further investigation.

In summary, we demonstrated a clinically and radiologically practical application of deterministic tractography to DTI data to evaluate the NSP pathway in PD, and detected decreased NSP connectivity in PD without requiring complex imaging sequences for anatomical localization or segmentation software. Our findings provide impetus to better model diffusion parameters for small fibre tractography in further larger, prospective longitudinal MR study in PD.

## Methods

Institutional ethics approval and written subject consent from subjects were obtained. The methods were carried out in accordance with the approved guidelines and regulations, and all experimental protocols were approved by Singhealth ethics committee. Forty subjects (21 PD patients and 19 healthy controls) had structural brain MRI and DTI scans on a 3Tesla MR scanner^8^, and were scanned in the “on”state to minimise movement artefact. PD patients were diagnosed by a movement disorders neurologist in a tertiary referral centre based on the United Kingdom PD Brain Bank clinical criteria. Controls were age- and gender-matched individuals without neurological conditions. Exclusion criteria were: 1. Clinical evidence of atypical features including supranuclear gaze palsy, autonomic dysfunction or cerebellar dysfunction, 2.PD patients who were wheelchair bound with severe disability, 3. Subjects with organ dysfunction or life threatening diseases, 4. Subjects who have contraindications to MRI study. Motor disability was charted with the Unified Parkinson’s Disease Rating Scale (UPDRS) during the “on” period. The diffusion data was acquired using a spin-echo echo planar imaging sequence with 30 non-collinear directions; b-values of 0 and 800 s/mm^2^; TE/TR = 86/8200 ms; 1.875 × 1.875 × 2 mm^3^ voxel size). Structural sequences were reviewed to exclude pathology in the regions of interest.

Deterministic brute-force tractography was performed using Diffusion Toolkit (trackvis.org, Version 0.6.2.2) on DTI images after upsampling with trilinear interpolation to 1 mm^3^ resolution in MATLAB (The Mathworks, Inc., version 7.9.0, Natick, Massachusetts, United States) using SPM8 (Wellcome Department of Imaging Neuroscience, London, United Kingdom), based on published protocol^11^. This is a user-friendly cross-platform software^14^,^15^ that allows interactive track data manipulation in real-time. Tracking was performed using the modified Fibre Assignment by Continuous Tracking algorithm, FA threshold of 0.2^16^,^17^, angle threshold of 60°^18^, and default step length of 0.1 mm, which were found to produce the most anatomically accurate reconstruction. Following brute-force tract reconstruction, two independent raters manually isolated the NSP using the tract visualization program TrackVis (trackvis.org, Version 0.5.2). This includes a tool for measuring point-to-point statistics along the length of the tract from a specific point and extraction of statistics from a particular section of the tract. A 3 mm-diameter disk-shaped ROI was placed in the ventral SN, which appears green for antero-posterior orientation on the standard color-coded DTI FA scan. Nigrofugal tracing has shown that nigrostriatal projections traverse the GP to synapse directly in the striatum^19^. In addition, striatomesencephalic fibers from the posterior putamen have been found to form a discrete bundle coursing through the GP to converge on the substantia nigra^18^. Hence, our second “target” 4 mm-diameter ROI sphere was placed in the ipsilateral inferomedial GP to isolate the NSP tract complex (Fig. 1). A 4-mm sphere allowed full capture of the fibres of interest, due to their tendency to spread slightly at the GP.

Most of the tracts isolated extended beyond the SN and GP ROIs into the brainstem and cerebral cortex probably due to limitations in the resolution of the DTI data, causing the algorithm to incorporate other tracts close to its ends as part of itself. Since our primary interest was the connectivity of the SN to the GP and putamen and to avoid dilution of any effects by extraneous, pathologically-uninvolved tracts, the tracts isolated were segmented by identifying the tract midpoint^11^, and taking the length of NSP tract complex no more than 15 mm proximal and 15 mm distal to the midpoint. Point-to-point profiles of the DTI parameters – FA, MD, AD, RD—along the defined tract segment and streamline count were extracted using the TrackVis Mean-versus-Distance function. Volume-averaged DTI parameters for the entire tract segment were computed with MATLAB. Results were analysed using the average of the left and right tracts.

Statistical analysis was performed using SPSS (Version 20.0) and R (Version 3.0.0). Intraclass correlation coefficient (ICC) values comparing the tract parameters obtained by the two raters were calculated using a two-way mixed model with absolute agreement and a confidence interval of 95%. The gender in PD and control groups was compared with Chi-square test, and the age with Student’s t-test. Averaged DTI parameters and streamline count of the NSP complex in PD and control groups were compared using Student's t-test, and correlated with UPDRS motor score using Pearson's correlation. The midpoint of the tract profiles was selected for univariate logistic regression analysis since it had were no missing values across subjects and was probably less susceptible to contamination by intersecting fibres. As there was no significant asymmetry in the DTI variables or in the motor symptoms between left and right, the values of the left and the right tracts were averaged.

## Additional Information

**How to cite this article**: Tan, W.-Q. *et al.* Deterministic Tractography of the Nigrostriatal-Nigropallidal Pathway in Parkinson's Disease. *Sci. Rep.* **5**, 17283; doi: 10.1038/srep17283 (2015).

## Figures and Tables

**Table 1:** Subject Demographics and Clinical Data.

Mean ± SD (range)	Control	PD	P Value
Number	19	21	
Number with NSP tracked	17	18	
Sex (M/F)	14/3	14/4	0.735
Age (yrs)	71.2 ± 5.2	72.2 ± 4.5	0.529
Disease duration (yrs)	NA	5.1 ± 2.9	
UPDRS MotorScore	NA	44 ± 15	
FA	0.452 ± 0.022	0.426 ± 0.023	**0.002**
MD (10^−3^ mm^2^/s)	0.79 ± 0.05	0.82 ± 0.04	**0.044**
AD (10^−3^ mm^2^/s)	1.19 ± 0.07	1.21 ± 0.06	0.352
RD (10^−3^ mm^2^/s)	0.59 ± 0.04	0.63 ± 0.04	**0.008**
Streamline count	57.9 ± 14.2	29.4 ± 8.6	** < 0.001**

Legend: NSP-nigrostriatal and nigropallidal pathway; UPDRS-Unified Parkinson’s Disease Rating Scale; FA-fractional anisotropy; MD-mean diffusivity; AD-axial diffusivity; RD-radial diffusivity.

**Table 2:** Intraclass coefficient (ICC) values for averaged DTI parameters and streamline count.

	ICC Value (95% CI)
FA	0.91 (0.86–0.94)
MD (10^−3^ mm^2^/s)	0.83 (0.74–0.90)
AD (10^−3^ mm^2^/s)	0.82 (0.72–0.89)
RD (10^−3^ mm^2^/s)	0.85 (0.76–0.90)
Streamline Count	0.91 (0.86–0.94)

*Two-way mixed model, absolute agreement.

Legend: FA-fractional anisotropy; MD-mean diffusivity; AD-axial diffusivity; RD-radial diffusivity.

**Table 3:** Univariate logistic regression comparing tract statistics at the tract midpoint between PD and control groups.

Independent Variable	B Estimate	SE B	Odds Ratio (95% Confidence Interval)	p-value
FA (10^−1^)	−1.01	0.53	0.36 (0.11–0.96)	**0.06**
MD (10^−4^ mm^2^/s)	0.66	0.449	1.94 (0.85–5.14)	0.141
AD (10^−4^ mm^2^/s)	−0.53	3.19	0.59 (0–333)	0.868
RD (10^−4^ mm^2^/s)	0.91	0.453	2.5 (1.1–6.8)	**0.0433**
Streamline Count	−0.24	0.087	0.78 (0.62–0.90)	**0.007**

Legend: FA-fractional anisotropy; MD-mean diffusivity; AD-axial diffusivity; RD-radial diffusivity.

## References

[b1] Damier P., Hirsch E. C., Agid Y. & Graybiel A. M. The substantia nigra of the human brain. II. Patterns of loss of dopamine-containing neurons in Parkinson’s disease. Brain. 122, 1437–1448 (1999). 10430830 10.1093/brain/122.8.1437

[b2] Kordower J. H. *et al.* Disease duration and the integrity of the nigrostriatal system in Parkinson’s disease. Brain. 136, 2419–2431 (2013). 23884810 10.1093/brain/awt192 PMC3722357

[b3] Whone A. L., Moore R. Y., Piccini P. P. & Brooks D. J. Plasticity of the nigropallidal pathway in Parkinson’s disease. Ann Neurol. 53, 206–213 (2003). 12557287 10.1002/ana.10427

[b4] Cochrane C. J. & Ebmeier K. P. Diffusion tensor imaging in parkinsonian syndromes: a systematic review and meta-analysis. Neurology. 26, 857–864 (2013). 23439701 10.1212/WNL.0b013e318284070c PMC3598454

[b5] Cercignani M. Strategies for patient-control comparison of diffusion MR data. Jones D. K. editor. Diffusion MRI: Theory, Methods, and Applications. Oxford University Press, Oxford. 485–499 (2010).

[b6] Johansen-Berg H. & Behrens T. E. J. Just pretty pictures ? What diffusion tractography can add in clinical neuroscience. Curr Opin Neurol 19, 379–385 (2006). 16914977 10.1097/01.wco.0000236618.82086.01 PMC3119814

[b7] Mukherjee P., Chung S. W., Berman J. I., Hess C. P. & Henry R. G. Diffusion tensor MR imaging and fiber tractography: technical considerations. Am J Neuroradiol. 29, 843–52 (2008). 18339719 10.3174/ajnr.A1052 PMC8128579

[b8] Chan L. L. *et al.* Transcallosal diffusion tensor abnormalities in predominant gait disorder parkinsonism. Parkinsonism Relat Disord 20, 53–59 (2014). 24126023 10.1016/j.parkreldis.2013.09.017

[b9] Menke R. A. *et al.* MRI characteristics of the substantia nigra in Parkinson’s disease: a combined quantitative T1 and DTI study. Neuroimage. 47, 435–441 (2009). 19447183 10.1016/j.neuroimage.2009.05.017

[b10] Sharman M. *et al.* Parkinson’s disease patients show reduced cortical-subcortical sensorimotor connectivity. Mov Disord. 28, 447–454 (2013). 23144002 10.1002/mds.25255

[b11] Zhang Y. *et al.* Diffusion tensor imaging of the nigrostriatal fibers in Parkinson's disease. Mov Disord. 30, 1229–1236 (2015). 25920732 10.1002/mds.26251 PMC4418199

[b12] Yoshikawa K., Nakata Y., Yamada K. & Nakagawa M. Early pathological changes in the parkinsonian brain demonstrated by diffusion tensor MRI. J Neurol Neurosurg Psychiatry. 75, 481–484. (2004). 14966170 10.1136/jnnp.2003.021873 PMC1738942

[b13] Prakash B. D., Sitoh Y. Y., Tan L. C. S. & Au W. L. Asymmetrical diffusion tensor imaging indices of the rostral substantia nigra in Parkinson’s disease. Parkinsonism Relat Disord. 18, 1029–1033 (2012). 22705126 10.1016/j.parkreldis.2012.05.021

[b14] Wang R., Benner T., Sorensen A. G. & Wedeen V. J. Diffusion toolkit: a software package for diffusion imaging data processing and tractography. Proc. Intl. Soc. Mag. Reson. Med. 15, 3720 (2007).

[b15] Feigl G. C. *et al.* Magnetic resonance imaging diffusion tensor tractography: evaluation of anatomic accuracy of different fiber tracking software packages. World Neurosurg. 994, (September) 1–7 (2013). 10.1016/j.wneu.2013.01.004 23295636

[b16] Chen D. Q. *et al.* Three-dimensional *in vivo* modeling of vestibular schwannomas and surrounding cranial nerves with diffusion imaging tractography. Neurosurgery. 68, (4) 1077–1083 (2011). 21242825 10.1227/NEU.0b013e31820c6cbe

[b17] Hodaie M., Quan J. & Chen D. Q. *In vivo* visualization of cranial nerve pathways in humans using diffusion-based tractography. Neurosurgery. 66, 788–795 (2010). 20305498 10.1227/01.NEU.0000367613.09324.DA

[b18] Lehéricy S. *et al.* Diffusion tensor fiber tracking shows distinct corticostriatal circuits in humans. Ann Neurol. 55, 522–529 (2004). 15048891 10.1002/ana.20030

[b19] Gauthier J., Parent M., Lévesque M. & Parent A. The axonal arborization of single nigrostriatal neurons in rats. Brain Res. 834, 228–232 (1999). 10407122 10.1016/s0006-8993(99)01573-5

